# Efficacy of prescribed injectable diacetylmorphine in the Andalusian trial: Bayesian analysis of responders and non-responders according to a multi domain outcome index

**DOI:** 10.1186/1745-6215-10-70

**Published:** 2009-08-14

**Authors:** Emilio Perea-Milla, Luis Carlos Silva Ayçaguer, Joan Carles March Cerdà, Francisco González Saiz, Francisco Rivas-Ruiz, Alina Danet, Manuel Romero Vallecillo, Eugenia Oviedo-Joekes

**Affiliations:** 1 Research Support Unit, Hospital Costa del Sol, Ctra Nacional 340, km 187, 29603 Marbella, Spain; 2 CIBER Epidemiología y Salud Pública (CIBERESP), Spain; 3 National Center for Medical Science Information (INFOMED), 27 St N#110. Vedado, 10400 Ciudad de la Habana, Cuba; 4 Andalusian School of Public Health, Campus Universitario de Cartuja, Cuesta del Observatorio 4, Apartado 2070, 18080, Granada, Spain; 5 Andalusian Foundation for Drug Abuse Attendance (FADA), Avda. Hytasa Edf. Toledo II Planta 2 Oficina 3, 41006, Seville, Spain; 6 School of Population and Public Health, University of British Columbia & Centre for Health Evaluations and Outcomes, Providence Health Care, Vancouver, BC, Canada

## Abstract

**Background:**

The objective of this research was to evaluate data from a randomized clinical trial that tested injectable diacetylmorphine (DAM) and oral methadone (MMT) for substitution treatment, using a multi-domain dichotomous index, with a Bayesian approach.

**Methods:**

Sixty two long-term, socially-excluded heroin injectors, not benefiting from available treatments were randomized to receive either DAM or MMT for 9 months in Granada, Spain. Completers were 44 and data at the end of the study period was obtained for 50. Participants were determined to be responders or non responders using a multi-domain outcome index accounting for their physical and mental health and psychosocial integration, used in a previous trial. Data was analyzed with Bayesian methods, using information from a similar study conducted in The Netherlands to select a priori distributions. On adding the data from the present study to update the a priori information, the distribution of the difference in response rates were obtained and used to build credibility intervals and relevant probability computations.

**Results:**

In the experimental group (n = 27), the rate of responders to treatment was 70.4% (95% CI 53.2-87.6), and in the control group (n = 23), it was 34.8% (95% CI 15.3-54.3). The probability of success in the experimental group using the *a posteriori *distributions was higher after a proper sensitivity analysis. Almost the whole distribution of the rates difference (the one for diacetylmorphine minus methadone) was located to the right of the zero, indicating the superiority of the experimental treatment.

**Conclusion:**

The present analysis suggests a clinical superiority of injectable diacetylmorphine compared to oral methadone in the treatment of severely affected heroin injectors not benefiting sufficiently from the available treatments.

**Trial Registration:**

Current Controlled Trials ISRCTN52023186

## Background

Opioid addiction is a chronic relapsing disease that affects the lives of sufferers in very different ways [1]. Opioid-dependent people continue using these drugs despite the consequences for their health, legal situation, social integration and personal relations [2]. Opioid substitution therapies (such as methadone, buprenorphine or diacetylmorphine) are intended to reduce illicit opioid use, deaths, disease and crime, as well as to improve patients' health, quality of life and psychosocial integration. Therefore, the effectiveness of a treatment may be reflected in different areas of patients' lives and as a consequence a treatment can be evaluated in different ways.

Various studies have provided evidence of the effectiveness, safety, viability and cost-effectiveness of prescribing diacetylmorphine (DAM) for the treatment of long term opioid-dependent persons who have not benefited from other treatments [3-11]. DAM is currently prescribed, as a regular programme or in the context of a clinical trial, in six countries: the UK, Switzerland, the Netherlands, Germany, Spain and Canada [12].

In the Dutch trial testing co-prescribed diacetylmorphine vs. methadone for long-term opioid dependence, treatment effectiveness was evaluated by means of a multi-domain outcome index (MDO) in order to obtain an overall measure of treatment success or failure [10,13]. The goal of the MDO is to assess response by means of a dichotomous variable addressing, as a combined measure, different aspects involved in the process of stabilizing drug-dependent patients: their physical and mental health and psychosocial integration.

It has been remarked that although a MDO allows to capture the complexity of drug-dependence and summarizes various measures by means of a single index, it does not enable the weighting of each dimension making difficult to evaluate in which particular aspects the patient has improved; moreover, a MDO makes it more complicated to perform comparisons with other studies [14,15]. The first of these problems may be addressed by separating the dimensions constituting the MDO, in order to determine their individual performance, as we have done in a previous analysis [11]. The goal of the present study is to overcome the second obstacle: we seek to evaluate the results of the DAM prescription trial carried out in Andalusia (Spain) with the multi-domain dichotomous index proposed in the Dutch study [10]. Here we analyze data from the Andalusian study by formally applying prior empirical evidence reported on the evidence of this treatment. In addition, we discuss the contribution of the results to the state of the art.

## Methods

We analyzed data from a randomized controlled trial comparing injectable DAM vs. oral MMT conducted in Andalusia, Spain, from February 2003 to December 2004. Study design, methods and results have been published elsewhere [11]. Briefly, 62 long-term, opioid dependent individuals with severe health and other drug related problems were randomized to receive either injectable DAM (plus oral methadone) or oral methadone alone. A total of 44 participants completed the 9 month treatment period and 50 completed the follow-up evaluations.

For the present study we analyse data from the Andalusian trial using a multi-domain outcome measure reported in a previous study conducted in The Netherlands, also comparing injectable DAM and oral MMT [10]. The MDO is a dichotomous index, imputing success when the patient shows at least 40% improvement at 9 months, compared to the baseline values, in physical health (MAP-H) [16], or mental status (SCL-90)[17], or social functioning (illegal activities and/or contact with non drug users), without a deterioration superior to 40% in any of these dimensions and no substantial increase (20%) in cocaine use. More details about this MDO can be found elsewhere [13].

Statistical analyses were performed using a Bayesian approach in order to take advantage of previous information, a strategy highly appropriated when working with small sample sizes (small samples are very common in trials aimed at treating conditions with low-incidence in the community). Previous information in big samples would have virtually no impact in the results. We calculated success rates, the relative risk (RR) and the respective 95% confidence intervals (CI). Using data derived from the Dutch study, *a priori *information was obtained for analysis of the Andalusian study data using Bayesian methods [18-21]. Analyses were performed by intention to treat, with no imputation for missing values. We denote by *θ*_1 _the percentage of patients who responded to the experimental treatment (DAM), while *θ*_2 _represents the percentage of those responding to the conventional treatment (methadone). Bayesian analysis enables us to calculate the probability of *θ*_1 _being greater than *θ*_2 _by a specified magnitude, based on the data from our trial and prior information from the Dutch trial. Upon clinical judgment and based on the target populations (i.e. treatment-refractory opioid-dependent individuals) and outcome expectations (i.e. stabilization, long-term treatment), we assumed as clinically relevant a minimal difference of 15% between the rates of responders in each group, and assessed the probability of this being fulfilled under different assumptions.

For each of the parameters *θ*_1 _and *θ*_2 _we selected three *a priori *distributions from the family of beta distributions with parameters **a **and **b **which approximately represent the implicit number of responders and non-responders in the prior distribution. These three scenarios represent different degrees of incorporation of prior evidence. In the first scenario ('No use' of historical data) Jeffreys' priors were used, which are non-informative prior beta distributions with parameters **a **= **b **= 0.5 for both, *θ*_1 _and *θ*_2_. The remaining two pair of priors were set on the basis of the knowledge derived from a previous clinical trial using injected DAM. [10] The respective CI associated with these prior data were calculated, and parameters were chosen (**a **and **b **in the beta distribution) such that the maximum density intervals of these distributions coincided approximately with the CI obtained previously. The second scenario ('Partial use') down-weighted the Dutch study by dividing **a **and **b **by 5. Finally, we repeated the process using the values **a **and **b **without modification ('Full use'), essentially equivalent to a full pooling of the trial results in a meta-analysis.

In order to perform a sensitivity analysis, several scenarios need to be imagined. The one considered when we do a 'partial use' of previous data is placed between two extreme situations: no use of previous data (meaning there are no similarities between contexts) and full use of them (meaning both contexts are equal). These extreme positions are extreme, since we cannot assume the Dutch and Andalusian context are the same, or that they have nothing in common either. The chosen halfway scenario takes into account this argument. A division by 5 of the parameters derived from the Beta-distribution was chosen in order to substantially increase the distribution dispersion attributed to previous data, allowing an adequate sensitivity analysis.

For each one of these prior choices, we obtained the conjugate beta distributions for the response rate in each arm of the trial using our binomial data. A total of 20.000 simulations were made from these *a posteriori *distributions, and the corresponding 20.000 differences *θ*_1 _- *θ*_2 _were calculated providing an *a posteriori *distribution of the difference between the proportions: Δ = *θ*_1 _- *θ*_2_. This was used to derive simulation-based estimates of the probability of relevant magnitudes concerning Δ: P(Δ larger than 0), P(Δ larger than 0.15) and a maximum density interval (probability interval for Δ) at 95%. EPIDAT 3.1 was used for all computations [22].

## Results

The *a priori *beta distributions, as stated above, were obtained using the data from the Dutch clinical trial. This was carried out with a sample of 98 patients in the experimental group (injectable DAM) and 76 in the control group (oral methadone). Twelve month success rates of 56% and 31%, respectively, were obtained. To define the above-mentioned informative *a priori *distributions, we began by calculating the 95% CI (frequentist) associated with the preceding data. Confidence intervals for the percentage of patients who responded to treatment in the control and experimental groups in the Dutch trial were (46-66) and (21-41) respectively; and the *a priori *beta distributions consistent with them were a_1 _= 55, b_1 _= 43, and a_2 _= 24, b_2 _= 52 respectively. Following the steps described in the methods section, the analysis was performed for the three possible scenarios, as described in Table 1.

**Table 1:** a and b values for each parameter *θ*_1 _and *θ*_2 _among the three groups of the a priori distributions used.

**Dutch information**	***θ*_**1**_**		***θ*_**2**_**	
	**a**_**1**_	**b**_**1**_	**a**_**2**_	**b**_**2**_

No use	0.5	0.5	0.5	0.5

Partial use	11.0	8.6	4.8	10.4

Full use	55.0	43.0	24.0	52.0

Among the patients in the experimental group (n = 27), the rate treatment responders was 70.4% (95% CI 53.2-87.6), while for those in the control group (n = 23) it was 34.8% (95% CI 15.3-54.3). The difference in response rates between the two groups was 36.6% in favour of the experimental group. The probability of a positive response to treatment by participants allocated to experimental group (RR) was 2.2 times greater than for those of the control group (95% CI 1.2-4.3; p = 0.012). The number needed to treat was 2.8 (IC 95% 1.6-10.0).

**Figure 1:**
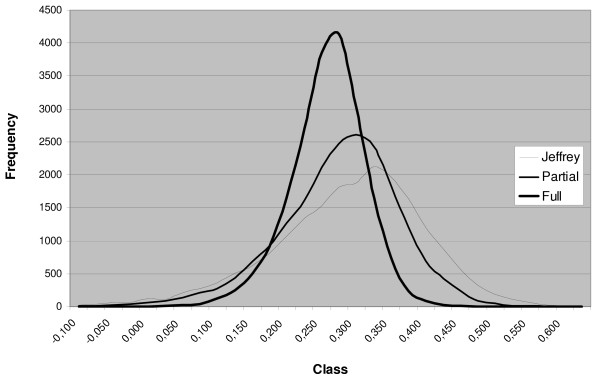
**Non parametric distribution of success rates differences between the experimental and control groups for the three possible scenarios (without using the Dutch data in order to determine the priors with partial and total use)**.

After using the data from the present study to update the *a priori *information, the nonparametric distributions obtained from the simulated differences in success rates (experimental less conventional) in the 3 scenarios is shown in Figure 1. This shows that the probability of success in the experimental group is higher than in the control group. In the last two cases, the whole distributions are located to the right of the zero, above the 6% level; in the first one, the distribution includes a very small fraction of negative values. The 95% probability intervals for the difference and probabilities of Δ >0 and Δ >0.15 are presented in Table 2.

**Table 2:** Probability values of the difference in success rates between the experimental and control groups being bigger than 0 and 0.15, and probability intervals (95%) for the possible three scenarios: without using the Dutch data in order to determine the priors, partial use, and total use).

Dutch information	P(Δ>0)	P(Δ>0.15)	Probability Interval **(95%)**
No use	0.994	0.926	8.0 - 57.6

Partial use	0.998	0.934	9.9 - 50.4

Full use	1.000	0.965	13.9 - 39.3

## Discussion

Our analysis of the Andalusian trial data using a multi-domain outcome measure as a treatment response criterion shows that the group receiving injectable diacetylmorphine had a greater probability of responding to treatment than the group that receive only oral methadone, both in clinical and in statistical terms.

The results obtained with this MDO are remarkable given that this indicator has a high level of exigency, as much by its complex definition, the magnitude of the demanded change (40%) and by the inclusion of the criterion of the cocaine consumption. Also, the MDO is a dichotomous variable, being less sensitive to change than the dimensional measures. For a fixed sample size a binary outcome measure would be able to detect a change of a 10% of the variance, whereas a dimensional measurement could detect changes of 1% [23].

It is important to note that the results come from a small sample and this limits their generalizability; other limitations derived from the design of the study have been discussed elsewhere [11]. When comparing the present study with the one conducted in the Netherlands [10], it should be taken into account that the control group in the Andalusian trial received larger average doses of methadone, and also they received an optimized version of MMT (involving greater psychosocial resources than the treatment that is normally provided). Also, the intervention lasted 12 months in the Dutch RCT, and 9 months in the Andalusian one. Nevertheless, the differences between the groups in the Dutch RCT stabilized after approximately 10 months.

In the present study the Bayesian analysis reveals a clear superiority of the diacetylmorphine-based treatment over methadone. The fact that the probability of the experimental treatment surpassing the conventional one by at least 15% gives such a high result (over 0.9 in the different scenarios) is important, especially considering the case in which this value is derived from the formal integration of earlier data with those from the present study. Our findings fit in with the *a priori *probability of the superiority of injectable DAM versus oral methadone in the case of treatment-refractory patients, and show how even partial use of the historical data reinforce the confidence in a clinically relevant difference.

The results obtained using Bayesian analyses are similar to those derived from the classical statistical approach when large sample sizes are used. The Bayesian method used in this analysis, however, was especially well suited because of the small sample size in our trial; in addition, it allowed to integrate previously obtained results into the current study to a partial or full extent. Moreover, this method is in agreement with recommendations of paying special attention to calculating the magnitude of the effect of the treatment being studied, and not so much on its statistical power [24,25].

## Conclusion

National and European data shows a stabilization in the use of heroin. However, a sub-group of heroin users with high health and social needs are not properly served by the health care system. Pharmacological alternatives are needed to attract and engage these individuals in treatment. The evidence for the greater efficacy of injectable DAM, in comparison with oral methadone, in the case of long-term, treatment-refractory opioid-dependent patients is supported by the present study and by others [4-6,10,26,27]. The next step would be to design a study evaluating the provision of DAM in standard clinical practice, i.e. in more ecological settings. However, the delay in the approval of those programs still depends more on the political and moral contexts than on the scientific conclusions reached over recent years [12,28].

## Competing interests

The authors declare that they have no competing interests.

## Authors' contributions

EPM, EOJ, JCM, MRV and FGS designed the study and gathered the data. The senior statistician (LCS) performed the data analyses. EPM, LCS and EOJ wrote the first draft of the paper and all authors contributed to the final version. The final decision about publishing the paper was made by all the authors. All authors vouch for the accuracy of the data and analysis.
